# Editorial: Blood Flow Restriction: Rehabilitation to Performance

**DOI:** 10.3389/fphys.2021.566421

**Published:** 2021-04-30

**Authors:** Stephen D. Patterson, Jamie F. Burr, Stuart Warmington

**Affiliations:** ^1^St Mary's University, Twickenham, United Kingdom; ^2^University of Guelph, Guelph, ON, Canada; ^3^Deakin University, Burwood, VIC, Australia

**Keywords:** Pre-conditioning–RIPC, occlusion, BFR, IPC, blood flow restriction

## Introduction

The manipulation of limb blood flow via the use of specialized cuffs, bands, or tourniquets may be used to target specific acute physiological responses or chronic training induced adaptations affecting strength, hypertrophy, or aerobic exercise efficiency and performance. This manipulation may be broadly captured by the techniques of (a) blood flow restricted exercise, and (b) ischemic pre-conditioning, and as such these form the basis/focus of this Research Topic.

Blood flow restriction (BFR) exercise is a relatively novel training technique used for increasing skeletal muscle mass and strength. This BFR technique restricts muscle blood flow through the application of an external pressure, typically using a pneumatic tourniquet/cuff system applied to the most proximal section of the upper or lower limbs. Inflation of the cuff produces a mechanical compression of the underlying tissues leading to a full or partial restriction of both venous and arterial vasculature. This variable degree of blood flow restriction likely affects a reduction in venous return while creating tissue hypoxia distal to the cuff, with the magnitude of both these effects being modulated by the phase and intensity of muscle contractile activity or exercise. Importantly, the gains in skeletal muscle size and strength with BFR training have been typically demonstrated when using light exercise loads/intensities (e.g., 20–30% one repetition maximum; walking exercise), generating supporting evidence for BFR during voluntary resistance and aerobic exercise, and also passively without exercise. More recent research has also examined the combination of BFR with non-traditional exercise modalities such as whole-body vibration techniques and neuromuscular electrical stimulation. Despite a growing body of evidence in support of beneficial skeletal muscle outcomes as well as functional performance benefits in a range of populations, at present, there is no universal standard method for the application of BFR during exercise (Patterson et al.). Differences exist for cuff type and size, pressures used the method for determination of cuff pressure and the duration of BFR application. Its present uses include rehabilitation from injury and to improve aspects of athletic performance.

Ischemic preconditioning (IPC) is, by contrast, when a tourniquet/cuff is placed on either the arm(s) or leg(s) of participants prior to exercise performance, irrespective of the exercise modality. The application of an IPC protocol pre-activity, which typically includes cyclic occlusion of remote or local skeletal muscle tissue of the limb, may render skeletal muscle of athletes more resistant to fatigue due to similar muscular modifications demonstrated in a clinical setting. As such IPC is currently being assessed as to its validity as an ergogenic aid in both aerobic and anaerobic exercise performance (Incognito et al., 2016). Furthermore, evidence exists around the use of IPC to assist with recovery from exercise/fatigue and adaptation to training.

## Topic Content

This Research Topic accepted 21 articles for publication (18 original research papers, 1 systematic review, 1 review and 1 opinion) written by a total 127 contributing authors. Overall, this topic pools research focused on acute responses and adaptations to blood flow restriction training and ischemic preconditioning. Based on the numerous contributions to this Research Topic we have learned the following:

## Acute Responses of Blood Flow Restriction Training

When examining the acute muscle, metabolic and cardiopulmonary responses to blood flow restriction resistance exercise (BFR-RE), Ilett et al. demonstrated a progressive response with increasing applied restriction pressure during BFR-RE that aligned with reduced MVC torque, increased blood lactate and EMG, and reduced muscle oxygenation. From this, the authors concluded that a minimum “threshold” around 60% limb occlusion pressure (LOP) may be necessary for BFR-RE to affect changes in these oft-cited training variables that may be intrinsic for longer term BFR-RE training adaptation. Reis et al. further examined the effect of different relative applied restriction pressures on muscle oxygenation during BFR-RE. Even with light-load BFR-RE (20% 1-RM), all pressures tested (40, 60, 80% LOP) induced muscle microvascular deoxygenation, but only pressures above 60% LOP demonstrated severely restricted reoxygenation during the intervals between exercise sets. Taken together, these two studies suggest higher pressures (60–80% LOP) are needed to provide sufficient acute physiological stimulation that may translate into chronic adaptations when utilized during training.

Scott et al. compared the hemodynamic responses to low-load resistance exercise (LL-RE), low-load BFR-RE and unrestricted high-load (HL-RE) in older women. While cardiac measures were similar between HL-RE and BFR-RE (e.g., cardiac output, stroke volume), the authors demonstrated significantly higher blood pressures (systolic, diastolic, and mean arterial pressure) for BFR-RE compared with both HL-RE and LL-RE, and this vascular stress was greater when more muscle mass was used during the exercise (e.g., leg press vs. leg extension). This reinforces suggestions that caution be exerted when prescribing BFR-RE to certain at-risk populations. Aligned with these cardiovascular effects (Montgomery et al.) examined circulating endothelial progenitor cells (EPCs), which are a vasculogenic subset of progenitors that play a key role in maintaining endothelial integrity. They hypothesized that EPC mobilization may be augmented by the local hypoxia observed with BFR-RE. However, the authors found that BFR-RE impairs this expected rise in circulating EPCs in the post-exercise recovery period compared with non-BFR exercise. Taken together these two acute studies suggest some caution and certainly further research needs to explore the long-term hemodynamic and vasculature effects of BFR-RE.

## Adaptations to Training With Blood Flow Restriction Training

When examining the suitability of BFR for older populations, Cook and Cleary compared the effect of BFR-RE against HL-RE in older adults who were at risk of mobility limitations. Across a 12-week intervention with similar training volumes between the HL-RE and BFR-RE the growth in knee extensor (KE) and knee flexor (KF) CSA was similar. However, KE strength gains were greater in the HL-RE group, most likely due to the greater training load, while training repetitions were greater in the BFR-RE group. Interestingly, when this training load difference was eliminated in the KF, and in fact was “relatively” greater in BFR-RE, resulting in similar gains in KF strength across the intervention. Ultimately, the authors conclude that even in older adults, incorporating systematic load progression throughout training periods should be employed to maximize strength gains. This work is in agreement with previous evidence that suggests BFR-RE shows less gain in muscle strength compared to HL-RE (Loenneke et al., 2012; Hughes et al., 2017; Lixandrão et al., 2018), however the most up to data meta-analysis suggests there to be no difference for both strength and muscle mass (Grønfeldt et al., 2020). Therefore, as per the guidelines in Patterson et al. it is recommended to use BFR-RE under specific circumstances (e.g., post-operative rehabilitation, cardiac rehabilitation, inflammatory diseases, and frail elderly) with a progression back to HL-RE as the ultimate aim.

A number of other studies also examined the differences between high and low load training prescriptions compared to more traditional RE controls. To investigate BFR-RE in a “real world” situation by using whole body resistance exercise, rather than the short-term upper or lower body BFR-RE commonly used in previous research, Brandner et al. employed a 12-wk study with both a training and detraining component. Comparing BFR-RE, HL-RE, LL-RE, or a non-exercise control (CON) the whole body BFR-RE improved both lower- and upper-body strength. Interestingly, given the use of multiple upper- and lower-body exercises across each training session, collectively lasting ~45 min, these changes were similar to LL-RE, but both groups were still lower in comparison with HL-RE, with all three training groups being greater than CON. This raises a question as to whether long-duration sessions and multiple exercises within a training session dampen the effectiveness of BFR-RE. Following the additional detraining period, whole body strength remained significantly elevated for both BFR-RE and HL-RE, but only the HL-RE group remained higher than all other groups.

Using an innovative training design to examine the mechanisms driving strength gains and hypertrophy with RE, Biazon et al. compared the effects of high mechanical tension training protocols with and without BFR (HL-RE and HL-BFR-RE), and metabolically stressful training protocols induced with BFR [BFR-RE (light-load) and HL-BFR-RE (high-load)] on deoxyhemoglobin concentration, muscle cross-sectional area (CSA), activation, strength, architecture and oedema before and after 10 weeks of training, with BFR released between sets. The authors concluded that mechanical tension and metabolic stress seem to share the variance of the muscle hypertrophy response under high mechanical tension protocols, while metabolic stress seems to be the main mechanism responsible for muscle hypertrophy when mechanical tension is low.

Aligned with this, Sieljacks et al. found that despite significantly greater strength gains with HL-RE (13–23%) compared with BFR-RE (6–10%), 6-weeks training produced greater increases in myofibrillar muscle protein synthesis, muscle RNA synthesis and total RNA content in both BFR-RE and HL-RE compared with a CON, with no significant differences between the two exercise groups. This study demonstrated that BFR-RE increased long-term muscle protein turnover and ribosomal biogenesis to a similar degree to HL-RE. Extending on this, Groennebaek et al. found that resistance exercise can stimulate mitochondrial biogenesis and respiratory function to support healthy skeletal muscle and whole-body metabolism. Intriguingly, BFR-RE produced similar mitochondrial adaptations at a markedly lower load, than HL-RE. Collectively, these studies support the clinical value of BFR-RE for populations in whom exercise with high loading is untenable.

Jessee et al. studied the effect of BFR-RE on the hypertrophic response to different load and pressure combinations of BFR-RE. They investigated muscular adaptations following resistance training with a very LL-RE alone (15% 1RM/0% LOP), with moderate BFR (15% 1RM/40% LOP), or with high BFR (15% 1RM/80% LOP), and compared them to HL-RE (70% 1RM/0% LOP). With the exception of 1RM, changes in strength and muscle size were similar, regardless of load or restriction. The training volume required to elicit these changes lowered with increased BFR pressure.

Due to the low load nature of BFR-RE there is a potential role of this mode of exercise in the pre-habilitation and rehabilitation of different injuries. Indeed, Žargi et al. demonstrated that short-term preconditioning with BFR-RE attenuated the deterioration of quadriceps muscle endurance in the postoperative period following ACL reconstruction. This enhanced quadriceps endurance was triggered by a combination of augmented muscle fiber recruitment and enhanced muscle perfusion. The latter alludes to a preserving effect of preconditioning with BFR-RE exercise on density and function of quadriceps muscle microcirculation within the first 4 weeks after ACL reconstruction. Alternatively, during the rehabilitation period Ladlow et al. evaluated the efficacy and feasibility of BF-RE training vs. HL-RE on the clinical outcomes of patient's undergoing inpatient multidisciplinary team rehabilitation for lower-limb injury. They found comparable improvements in muscle strength and hypertrophy between BFR-RE and HL-RE following in-patient rehabilitation. The BFR-RE group also achieved significant improvements in functional capacity, suggesting BFR-RE as a rehabilitation tool with the potential to induce positive adaptations without high mechanical loads. As such, BFR-RE could be considered a treatment option for patients suffering significant functional deficits for whom conventional loaded RT is contraindicated.

## Safety and Efficacy

Crisafulli et al. investigated the effect of safety with longer term (1 month) training with BFR-RE. While blood pressure and systemic vascular resistance remained unaltered at rest, the authors found that BF-RE reduced blood pressure during handgrip exercise, thereby suggesting a potential hypotensive effect of this modality of training. However, this reduction in MAP during handgrip exercise seemed not to be mediated by the metaboreflex, which remained unaffected following the training period. Clarkson et al. completed a systematic review to determine whether exercise interventions utilizing BFR were able to improve objective measures of physical function indicative of activities of daily living. Using data from 13 studies they report BFR exercise, including multiple modalities, improved objective measures of physical function indicative of activities of daily living.

The work by Patterson et al. brought together much of the current research from this Research Topic and the literature in general. The authors set out a series of guidelines for BFR exercise, focusing on the methodology, application, and safety of this mode of training to inform practitioners how BFR exercise should be applied. Included with this the authors not only set out guidelines for BFR-RE, but also BFR with aerobic exercise and passive BFR, while providing clear guidance for the pressures, sets, reps etc. that should be used when employing this technique. This work should be regularly updated to keep abreast of any changes and updates in the literature.

## Ischemic Preconditioning

The ability of ischemic preconditioning (IPC) to enhance exercise capacity may be mediated through altering exercise-induced blood flow and/or vascular function. To this end, Cocking et al. demonstrated that a single local-IPC (but not remote-IPC) performed 20 min prior to a 30 min handgrip exercise (25% MVC) enhanced dilation of the exercise-induced conduit artery diameter (brachial artery). However, this change did not in fact translate into increased blood flow during exercise, nor did it impact post-exercise vascular function (via FMD). However, when using repeat applications of IPC, Jeffries et al. studied the impact on metabolic and vascular adaptations. Following 7 days of repeated IPC the authors showed skeletal muscle oxidative capacity and microvascular muscle blood flow to be increased. These findings are consistent with enhanced mitochondrial and vascular function following repeated IPC and may be of clinical or sporting interest to enhance or offset reductions in muscle oxidative capacity.

Slysz and Burr were interested to see if the ergogenic efficacy of ischemic preconditioning (IPC) increased when combined with greater tissue level oxygen consumption and metabolite accumulation as a result of concurrent light intensity muscle contraction under IPC. Using participants for whom a traditional IPC stimulus was not effective, each underwent four experimental conditions in a cross-over experimental design: (i) no IPC control, (ii) traditional IPC, (iii) IPC with EMS, and (iv) IPC with treadmill walking. For conditions where the IPC stress was magnified with the addition of muscle contractions while under occlusion, participants demonstrated a subsequent enhancement of the exercise performance response. These findings support the amplification of the ischemic preconditioning stimulus to augment the effect on exercise capacity.

Interested to see if IPC could help with recovery from repeated sprint exercise, Lopes et al. found that IPC did not change long-term heart rate recovery or heart rate variability throughout recovery, nor did IPC change any energy metabolism parameter. In conclusion, IPC accelerated short-term recovery to some extent, but did not change the long-term recovery of cardiac autonomic control from RSE, and such accelerator effect was not accompanied by any IPC effect on surrogates of energy metabolism responses to repeated sprint exercise.

Preconditioning may be performed with other modalities not just IPC. Thijssen et al. examined the impact of 12-week continuous training vs. high-intensity interval training on brachial artery endothelial ischaemia/reperfusion-injury in heart failure patients. They found that 12-week exercise training in heart failure patients mitigated endothelial ischaemia-reperfusion injury, an effect independent of the type of exercise. These changes may contribute to the cardioprotective effects of exercise training, whilst highlighting the potency of exercise as a pre-conditioning stimulus.

The opinion piece by Marocolo et al. listed some methodological concerns about protocol design, data analysis, and interpretation, of IPC research. They suggested the need for future studies to test shorter protocols (e.g., 2 × 2–3 min occlusion/reperfusion), which are more time-efficient (e.g., 8–12 min vs. 40 min) and more easily inserted in real-world settings of athletes/competitions if positive and meaningful findings are confirmed. Also, testing treatments controlled by different cuffing pressures (i.e., SHAM, IPC, and no cuff—control) should assess the effect of IPC on higher fitness subjects (i.e., elite athletes). Only then may we be able to draw robust conclusions as to whether IPC is suitable for recreational practitioners and/or elite athletes.

## Conclusion

Taken on whole, the evidence produced from papers in this Research Topic highlight a number of potential roles for the use of blood flow manipulation to positively affect both health and human performance across the lifespan. While blood flow manipulation does appear safe and effective for increasing protein synthesis, muscle hypertrophy, and strength compared to non-restricted light-load exercise, the precise mechanisms of these changes still require characterization. Further optimization of these procedures with regard to the timing, pressure, periodization of use, and with regard to client/athlete training goals will help to guide its targeted and evidence-based use. The application of IPC appears to have the ability to alter exercise performance through pathways associated with local muscle perfusion and metabolism. Similar to BFR, the work herein sheds light on some potential mechanisms underlying the observed effects, but our ability to use these techniques in clinical or performance settings would benefit from further mechanistic work and an understanding of how these protocols are optimized when accounting for the interactions of participant characteristics, exercise parameters, and IPC stress. Future work should aim to address some of the limitations listed above for both BFR and IPC research as the current evidence in this Research Topic only sheds a limited light on the current area.

## Author Contributions

All authors listed have made a substantial, direct and intellectual contribution to the work, and approved it for publication.

## Conflict of Interest

The authors declare that the research was conducted in the absence of any commercial or financial relationships that could be construed as a potential conflict of interest.
